# An Overview of Nutritional Interventions in Inflammatory Bowel Diseases

**DOI:** 10.3390/nu16183055

**Published:** 2024-09-10

**Authors:** Ramit Magen-Rimon, Andrew S. Day, Ron Shaoul

**Affiliations:** 1 Pediatric Gastroenterology & Nutrition Institute, Ruth Children’s Hospital of Haifa, Rambam Health Care Campus, Faculty of Medicine, Technion—Israel Institute of Technology, Haifa 3525408, Israel; r_shaoul@rmc.gov.il; 2 Department of Paediatrics, University of Otago Christchurch, Christchurch 8011, New Zealand; andrew.day@otago.ac.nz

**Keywords:** inflammatory bowel diseases, pediatric, nutrition, diet, Crohn’s disease, ulcerative colitis

## Abstract

Food is an important environmental factor in the development of inflammatory bowel diseases, chronic immune-mediated diseases of the gastrointestinal tract. Consequently, there is significant focus on the role that dietary approaches might have in the management of these diseases. The introduction of exclusive enteral nutrition (EEN) as a treatment option for induction of remission in Crohn’s disease was a breakthrough in disease pathophysiology understanding and has paved the way for dietary options based on this understanding. This review aims to summarize the current data on the effect of different available diets on disease symptoms and the inflammatory process.

## 1. Introduction

The conditions known as inflammatory bowel diseases (IBDs) are chronic inflammatory diseases that primarily involve the gastrointestinal tract [1]. The two main types of IBDs are Crohn’s disease (CD), where the inflammatory process can involve any part of the intestinal tract, and ulcerative colitis (UC), where the inflammation is limited to the colon.

While the pathophysiology of these diseases is not completely understood, interactions between the intestinal microbiome and the immune system, triggered by environmental factors in a person with underlying genetic risk factors, are considered to be critical [1]. Strong epidemiological data indicate the importance of diet and dietary patterns as environmental triggers, with particular regard to so-called Westernized diet choices implicating industrial and ultra-processed foods and other components [2].

Over the past few decades, there has been increasing focus from both patients and healthcare professionals on the potential role of diet in the management of IBDs. Individuals with IBDs tend to change their diet after diagnosis due to their beliefs and experiences in order to reduce gastrointestinal symptoms and control intestinal inflammation [3,4,5].

Coincident with this increasing interest, a number of novel dietary approaches for either the induction or maintenance of remission have been developed and assessed [6,7,8,9,10]. These include dietary approaches that involve using liquid nutritional products or restrictive diets where various components are eliminated. The objective of this review is to summarize current nutritional approaches and to review the available data on their efficacy in the treatment of CD or UC in children or adults.

## 2. Exclusive Enteral Nutrition (EEN)

### 2.1. EEN in Crohn’s Disease

EEN is a dietary approach that involves the ingestion of a liquid formula as the sole nutritional intake for a number of weeks. It is recommended in European guidelines as the first-line therapy for induction of remission in children with active CD [6] and is used widely in pediatric units [11,12]. Typically, EEN is used less frequently in adults with CD, but is widely used in countries such as Japan [13]. It has, however, been suggested to be considered as a therapeutic option in the setting of steroid intolerance in adults with CD [14].

The use of EEN has been associated with relatively few and minor adverse events, such as vomiting and diarrhea. Refeeding syndrome, notably a more significant complication, has been described in a few case reports [15,16]. These outcomes likely reflect the underlying nutritional state of the children rather than being specific to EEN.

In children with active CD, EEN has similar rates of clinical remission as those seen with corticosteroid therapy [6]. In addition, EEN is noted to lead to significantly higher rates of endoscopic and histologic remission (mucosal healing) compared to corticosteroids [17,18,19,20]. EEN has been demonstrated to be effective in inducing clinical, biochemical and endoscopic remission in adults with CD as well [21].

Various enteral formulae can be utilized for EEN. Elemental and polymeric formulae are comparable in terms of clinical remission rates [19], yet polymeric formulae are associated with superior weight gain after six weeks of treatment [22]. Generally, polymeric formulae exhibit much better oral tolerance and better taste characteristics than elemental preparations [23,24]. Interestingly, some liquid formulae used for EEN contain food additives (such as carrageenan and polysorbate) that have been considered to have pro-inflammatory effects [21,25]. The presence of these components does not appear to adversely impact the outcomes of EEN treatment [25].

Although there are a lack of definitive data to guide the ideal duration of a course of EEN, the European Society for Pediatric Gastroenterology, Hepatology and Nutrition (ESPGHAN) CD guideline recommends eight weeks of EEN for induction of remission [26]. Surveys suggest that most centers prescribe 6–8 weeks of EEN, but international data suggest a wide variation in duration ranging from 2 to 12 weeks [24,26,27].

Sigal Boneh et al. [28] demonstrated a rapid response and remission even after a short period of three weeks in a group of 73 pediatric patients with mild to moderate CD. As a result of this observation, a cycling approach has been suggested as a way to maintain remission by using short periods of EEN in a cyclic manner [29]. In this study, 112 children with CD that completed 6 weeks of EEN for induction of remission were randomized to a group that was treated with 2 weeks of EEN every 8 weeks and a group that received a normal diet with partial enteral nutrition (PEN) of 25% of the caloric intake without additional therapy. At one year of follow up, the group that received repeated cycles of EEN had better outcomes in terms of maintenance of remission and mucosal healing.

In addition to induction of remission, EEN has also been demonstrated to have a role in the management of complicated CD [30,31]. These reports entail small and larger case series and have focused on children or adults with strictures, enterocutaneous fistulas or penetrating disease associated with phlegmon.

The exact mechanism of action of EEN is not fully understood. The putative mechanisms of EEN include the following: (1) exclusion of food antigens, (2) improving nutritional deficiencies, (3) inducing a change in the gut microbiome, (4) improved barrier function and (5) direct anti-inflammatory effects [21,23,32]. The available data clearly indicate that the effects of EEN are broader than just the exclusion of complex foods. It is likely that the mechanism of EEN involves two or more of the suggested pathways working in concert.

One of the key observations in recent years has related to the significant changes in the patterns of the intestinal microbiome during a course of EEN. Whilst different techniques have been utilized across many studies, the overall outcome is a reduction in microbial diversity with EEN [7,33]. Clearly, this is somewhat paradoxical given the general dysbiosis noted in individuals with CD [34].

There are insufficient data to guide the reintroduction of food after a course of EEN. The ESPGHAN guideline recommends that food reintroduction should be gradual over 2–3 weeks [26]. Nevertheless, Faiman et al. [35] retrospectively compared the outcomes of a group of 20 children who were reintroduced to food over five weeks to a second group that returned to solid foods over just three days. The two groups had comparable relapse rates 6 and 12 months after diagnosis and similar duration of remission [35]. Repeated reintroduction of EEN has been suggested as a way to maintain remission (REF). Some of our patients prefer this method to avoid/delay the introduction of biologics and immunosuppressants. Nevertheless, compliance is problematic and rarely lasts long, according to our experience.

### 2.2. EEN in Ulcerative Colitis

Sahu et al. [36] conducted an open-label randomized controlled trial, in which patients who were admitted with acute severe UC were randomized 1:1 to EEN or standard of care (SOC). Patients on EEN received a semi-elemental formula for 7 days along with SOC. The primary outcome was corticosteroid failure, defined by the need for salvage via medical therapy or a colectomy. Of 62 patients (mean age 35.3 ± 12.1 years; 40% male), 32 were randomized to EEN and 30 to SOC. The corticosteroid failure rate was lower on EEN compared to SOC (intention-to-treat analysis 25% vs. 43%, *p* = 0.051; per protocol analysis 19% vs. 43%, *p* = 0.04), without any difference in colectomy rate (9% vs. 13%; *p* = 0.41). Patients on EEN had a shorter hospital stay [median (range) 10 (8–17) vs. 13 (8–24) days; *p* = 0.04], higher day-7 albumin level (34 ± 4 vs. 29 ± 3 g/L, *p* < 0.01), greater reduction in serum C-reactive protein and fecal calprotectin levels (both *p* = 0.04) and a lower composite outcome of colectomy/hospitalization at 6 months (16% vs. 39%; *p* = 0.045) compared to SOC.

EEN has been used in several animal models of colitis. Kajiura et al. [37] reported that an elemental diet significantly suppressed inflammation in an interleukin (IL)-10–deficient cell transfer mouse model of colitis. This reduction in inflammation observed with elemental diet was associated with a decrease in the abundance of *Lactobacillus* spp. and an increase in *Enterococcus* spp. Additionally, *Lactobacillus* and, to a much lesser extent, *Enterococcus* spp. isolates were subsequently shown to induce tumor necrosis factor-alfa (TNF-α) and IL-6 production in an in vitro model using a RAW 264 macrophage cell line.

Some earlier reports demonstrated that EEN may have benefits in individuals with UC [38]. A Polish study evaluated EEN in groups of children with CD and UC [39]. Although the precise response rate was not detailed for either group, the authors delineated improvement in inflammatory markers, improved symptoms and nutritional benefits in the subgroup with UC.

In addition, in an experiment using the dextran sodium sulfate model (which replicates UC), mice receiving EEN had less inflammatory changes and less weight loss than untreated animals [40]. In addition, a novel and optimized EEN formulation had greater benefits than standard polymeric formulae in these animals. Similarly, EEN had clear benefits in IL-10 knockout mice infected with *Helicobacter trogontum* as a bacterial trigger to induce colitis [41].

Bajaj et al. [42] have recently evaluated the impact of seven-day EEN-conjugated corticosteroid therapy in patients with acute severe UC (n = 20). Their work focused particularly on changes in the intestinal microbiome. The group that responded to EEN had enhanced abundances of beneficial bacterial genera *Faecalibacterium* and *Veillonella* and reduced abundance of *Sphingomonas* compared to non-responders. In contrast, no changes in the microbiome were observed in the SOC group (n = 24). The authors suggested that microbiome assessment may serve as a predictor of response to EEN in this population.

## 3. Crohn’s Disease Exclusion Diet (CDED)

In part due to concerns about adherence to a formula-based liquid-only diet for the induction of remission, there have been a number of solid-food-based diets developed in recent years. The CDED is a whole-food diet focused on the exclusion of particular foods or food groups considered to be harmful with the inclusion of foods considered to be safe [43]. The diet involves an initial phase comprising six weeks of dietary restrictions in conjunction with 50% of caloric intake provided by a polymeric formula. The second phase involves a further six weeks of restricted foods with 25% of caloric intake from a polymeric formula.

In a head-to-head comparison study involving 78 children with mild to moderately active CD, CDED was shown to be as effective as EEN in the induction of remission [7]. In this evaluation, CDED was also tolerated better than EEN. More recently, an initial open-label study has also shown that CDED can be effective in the induction of remission in adults with mild to moderate CD [44].

In addition, CDED has been demonstrated to be effective in the maintenance of remission for up to 24 weeks in adults with CD and up to 12 weeks in children with CD [7,44]. However, more studies are needed to explore the effectiveness of CDED in the maintenance of remission for longer periods.

## 4. Partial Enteral Nutrition (PEN)

PEN includes the use of a portion of the caloric intake as a formula combined with solid food. Whilst most data have indicated that PEN is substantially less effective than EEN for the induction of remission [45,46,47], one recent study has indicated potential benefits [48]. In a small pilot study conducted on children with mildly active CD, Urlep et al. [48] compared the outcomes of EEN to those of PEN. The protocol for PEN in this study comprised 75% of the caloric intake as formula with 25% as one solid meal each day based on an anti-inflammatory diet (AID). The clinical and endoscopic remission rates were similar in both groups of children. Similar observations were reported in an earlier report while using only 50% of caloric intake as a formula [7].

In contrast to the potential role of PEN for induction of remission, there are numerous studies indicating a role in the maintenance of remission in adults and children [10,44].

In addition, a meta-analysis by Yang et al. [49], which included eight small cohort studies of individuals with mild to moderately active CD, demonstrated beneficial effects of PEN in preventing clinical relapse during periods of 0.5–2 years as compared to not using PEN. These data support the use of PEN as an add-on treatment to the pharmaceutical therapy of CD.

## 5. CD-TREAT and Tasty and Healthy

CD-TREAT is a novel dietary approach that aims to imitate the effects of EEN on the microbiome using specific whole foods [8]. This diet involves the exclusion of putative pro-inflammatory food components (such as alcohol, lactose and gluten) and the inclusion of foods that are high in starch and low in fiber content.

In a pilot study involving five children with mild to moderate CD, an eight-week course of CD-TREAT resulted in clinical remission in 60% of the patients and a 55% reduction in fecal calprotectin [8]. In addition, as shown in 25 healthy adults, the effect of this diet on microbial composition and metabolome was similar to that seen after exposure to Modulen^®^-based EEN [8].

Tasty and Healthy, another dietary approach utilizing a whole-food diet, proposed and developed by an Israeli group, is being evaluated in an ongoing RCT (ClinicalTrials.gov ID NCT04239248). This diet excludes pro-inflammatory ingredients similar to the CD-TREAT diet and does not include a liquid formula, as opposed to the CDED.

These new diets are more palatable than the exclusively formula-based diet, hence the increasing adherence. The primary data are encouraging, but for now, according to the last published ECCO-ESPGHAN guidelines [6], these diets are still not recommended as a treatment in CD since more data and replication studies are needed.

## 6. Ulcerative Colitis Exclusion Diet (UCED)

The underlying concept of this diet is to exclude foods reportedly associated with the development of UC [9]. The UCED includes high-fiber, low-fat (specifically animal fat, saturated fat and polysaturated fat) and low-protein ingredients and excludes food additives. Similar to the CDED mentioned earlier, the first phase of the UCED extends over six weeks and includes mandatory foods. Individuals who respond to the first phase then progress to the second phase, which is less restrictive, over a further six weeks.

The UCED has been evaluated in one open-label study involving 24 children with mild to moderately active UC [9]. The clinical response rate at the end of the first phase was 70.8%, while the clinical remission rate was 37.5%. A total of 66% of the initial responders remained in clinical remission by the end of the second phase (12 weeks). Although a reduction in fecal calprotectin was noted between baseline and weeks 3–6, this change was not significant due to the small number of subjects.

The UCED has been evaluated in one other small study that involved 15 adults with refractory UC [9]. In this report, a 12-week UCED diet was compared to fecal microbial transplantation (FMT) with free diet with and without preconditioning of the donor with the UCED for 12 days. The UCED resulted in a clinical response of 40% compared to 11.8% with FMT after eight weeks of treatment. Endoscopic remission rates of 27% and 12%, respectively, were observed at the end of week eight. The sample size prevented further statistical analysis, so the study was discontinued.

These two small pilot cohorts suggest that this diet may have beneficial anti-inflammatory effects in patients with UC, albeit with lower response rates than those seen with CDED. Further larger clinical trials are required in order to explore this diet and to consider which patients might benefit more.

## 7. Specific Carbohydrate Diet (SCD)

This diet involves exclusion of all complex carbohydrates (grains, dairy products that contain lactose and sweeteners except honey) and permits only monosaccharides in order to prevent fermentation and bacterial overgrowth that might generate dysbiosis [50].

Suskind et al. [51] conducted a small study that included 10 children with mild to moderate CD and compared an SCD to more permissive diets. In total, four patients were on an SCD, four were on a modified SCD (rice and oats allowed) and four were on a whole-food diet (only requiring elimination of wheat, corn, sugar, milk and food additives). Although all groups achieved clinical remission at week 12, normalization of C-reactive protein (CRP) and a decrease in ESR was only observed in the SCD and modified SCD and not in the more permissive whole-food diet.

A clinical and biochemical response to the SCD was also demonstrated in a retrospective study in children with CD and UC [52]. Overall, 12 (46%) out of 26 patients (20 CD and 6 UC) achieved clinical remission accompanied with a biochemical response. Some of the patients were able to stop medication and maintained remission on this diet alone for periods of up to 48 months. However, the authors reported that the children had difficulty maintaining adherence to the SCD over long periods.

Another small study that included 10 children with CD, who had a mucosal response evaluation via video-capsule endoscopy, demonstrated a significant decrease in clinical scores (PCDAI and HBI) and Lewis score after 12 weeks of an SCD, with 60% achieving clinical remission [53]. Maintenance of clinical remission at week 52 was achieved in six of seven patients who continued the SCD. No changes were observed in biochemical values or weight after 52 weeks; however, an increase in the Lewis score was noted. Patients who were receiving medical therapy at recruitment did not need escalation while following this diet.

A series of 50 adults with IBDs (predominantly with CD) reported symptom resolution after a mean duration of 10 months of the SCD [54]. Patients self-reported 91% efficacy in achieving clinical remission and 92% in maintaining clinical remission. However, 40% of the cohort reported difficulty in maintaining the dietary changes. Although seemingly promising, there was no objective assessment of the effects of the SCD upon inflammatory markers or mucosal healing in this cohort.

Symptomatic remission was also reported in an online survey completed by 417 adults with IBDs (47% CD, 43% UC, 10% IBD-U) [50]. One-third of this group reported clinical remission after two months and 42% after 6–12 months of following the SCD.

## 8. Low-FODMAP Diet

There is high prevalence of functional gut symptoms (especially irritable bowel syndrome (IBS) in patients with IBDs [55]. A meta-analysis that included 12 studies reported that 36% of patients with UC in remission and 46% of those with CD reported IBS-like symptoms.

In a survey conducted by Cohen et al. [56] on 2329 patients with IBDs, the investigators emphasized that patients believed that the foods they eat have a major influence on their symptoms. Yogurt, rice and banana were recognized as improving symptoms while vegetables, spicy or fried food, fruits, nuts and beans, milk, red meat, popcorn, dairy, high-fiber foods, alcohol and coffee were reported as foods that lead to worsening symptoms [56].

The low-FODMAP diet (fermentable oligosaccharides, disaccharides, monosaccharides and polyols) was developed for patients with IBS and is based on the exclusion of small fermentable carbohydrates that may not be metabolized in the small bowel and that then undergo bacterial fermentation in the colon, resulting in symptoms such as bloating, abdominal pain and changes in stool consistency [57]. The low-FODMAP diet requires a period of exclusion of all FODMAPs followed by phased reintroduction of specific groups to identify triggers for that individual. Ongoing exclusion of all groups of FODMAPs is not generally recommended or warranted. Whilst this diet is difficult and restrictive initially (in the full exclusion phase), it would generally be less restrictive and more manageable in the long term.

The main role of the low-FODMAP diet in the setting of IBDs is to manage functional symptoms. Several studies conducted in adults with IBDs in remission or with mild disease have demonstrated a positive effect of this diet on gut symptoms [58,59,60]. One study did not show improved symptoms related to constipation [61], whilst another reported a positive impact on the quality of life of patients with CD in remission [60]. A case series of children with IBDs in remission managed with a low-FODMAP diet also showed beneficial effects on their functional gastrointestinal symptoms [62].

The effect of this diet on gut inflammation has also been assessed in two clinical trials. One RCT conducted on 27 patients with IBDs in remission but with IBS symptoms found no differences in calprotectin or CRP levels between patients on a control diet and those on a low-FODMAP diet at the end of four weeks [59]. However, these patients were in biochemical remission at enrolment. A larger study conducted on 55 patients with IBDs, mostly (70–85%) in remission or with mild disease, reported a significant decrease in FC levels after six weeks of a low-FODMAP diet, with no change seen in the control group having a regular diet [58].

## 9. IgG4-Guided Exclusion Diet

The underlying concept of this dietary approach is that foods that prompt a greater Immunoglobulin (Ig)-G4 response are more likely to trigger inflammation and that exclusion of such foods is beneficial [63]. This diet was assessed in one clinical trial involving 98 patients with quiescent or active CD. The IgG4 responses to 16 foods were assessed. The outcomes of patients (n = 50) with the four foods having the greatest IgG4 titers were compared to those of a second group (n = 48) who had the lowest IgG4 titers. Milk, beef, pork and eggs were the most commonly excluded foods from the high-titer group. Disease activity scores in the first group improved compared to those in the second, but there were no observed differences in inflammatory markers between the groups [63].

## 10. Anti-Inflammatory Diet (AID)

Olendzki et al. [64] introduced the IBD-AID in 2014. This dietary approach includes the limitation of certain carbohydrates, such as lactose and complex carbohydrates, increasing intake of pre- and probiotics, use of unsaturated fats such as omega 3 instead of trans- or polysaturated fats and limiting any specific foods that are not tolerated. Changes in food texture were also included with a reflection of the inflammatory phase (flare, remission, strictures) of each subject. Among the 27 patients that completed four weeks of this diet, 24 reported good or very good response to the diet defined by reduced symptoms. In total, 11 patients (8 CD, 3 UC) achieved de-escalation of their medical treatment after completing this diet. Nevertheless, changes in inflammatory markers or endoscopic response were not evaluated.

## 11. Mediterranean Diet

This diet includes intake of unsaturated and polyunsaturated fats instead of saturated fats and trans-fatty acids, and as the anti-inflammatory diet, vegetables, whole grains and low intake of red meat [50]. Adherence to this diet appears to reduce the risk of various chronic diseases and overall mortality [65]. It is also associated with lower levels of inflammatory markers in adults (including TNF-α and high-sensitivity CRP) [66].

Despite the clear overall support for this dietary pattern, the data regarding the impact of this diet on patients with IBDs are limited. Chicco et al. [67] have followed 142 patients with IBDs who were on this diet for six months and found a reduction in liver steatosis as well as an increased quality of life. It was also observed that fewer patients on this diet had active disease and elevated inflammatory markers at the end of a 6-month follow-up compared to recruitment, yet this these data were available for only a minority of the patients. Recently, a randomized trial on 191 adult patients with CD that compared an SCD to the use of a Mediterranean diet [68,69] found similar clinical remission rates for both diets (46% vs. 43%, respectively) with very limited biochemical responses seen (only about 5% for both diets) (Table S1 in Supplementary Materials). The authors concluded that the Mediterranean diet might be the preferred diet between the two due to easier adherence and additional health benefits, such as prevention of cardiovascular disease.

## 12. Discussion

It is clear that diet has an important role in the pathogenesis of IBDs, and also has therapeutic roles. Most diets evaluated in the setting of IBDs involve the exclusion of suspected pro-inflammatory ingredients, mainly milk, wheat and food additives, as well as high-fat meat.

It should be noted that most diets have beneficial effects on clinical symptoms, which may alleviate functional symptoms that were demonstrated to coexist in a considerable percentage of patients with IBDs, especially in patients with CD [55]. Nevertheless, only a few diets have been demonstrated to also have a positive effect on the inflammatory process, with a reduction in inflammatory mediators.

To date, various dietary intervention studies have been conducted on individuals with mild to moderately active IBDs, predominantly people with CD and with relatively small sample sizes. Consequently, the generalizability or specificity of these observations is unclear. Furthermore, no conclusion on the efficacy of these diets on the inflammation or complication prevention can be made.

EEN is the only proven dietary intervention that has been shown to be effective in the induction of remission in active CD. However, the limitations of having a purely liquid diet can compromise adherence. Partial enteral nutrition used in conjunction with other interventions does appear to enhance the maintenance of remission and could be considered for all patients with mild to moderately active CD. Further evaluations of PEN in other settings, such as those with moderate to severely active disease treated with biologics or small molecules, require further evaluation.

In conclusion, although diet clearly plays a key role in the development of IBDs and it is tempting to use a specialized diet as part of the treatment in these patients, clinical data on the efficacy of those dietary interventions on intestinal inflammation is limited. Further studies are required to more clearly elucidate the role of specific diets and food supplements not only in treating symptoms but as anti-inflammation treatments.

## Supplementary Materials

The following supporting information can be downloaded at: https://www.mdpi.com/article/10.3390/nu16183055/s1, Table S1. Data summary for different diets for pediatric and adult patients with IBD.
