# Supplementary material for: An Overview of Nutritional Interventions in Inflammatory Bowel Diseases
Source: Nutrients. 2024 Sep 10;16(18):3055. doi: 10.3390/nu16183055 (PMC11435346; doi:10.3390/nu16183055)
Supplement: Supplementary file 1 [file nutrients-16-03055-s001.zip › nutrients-3162235-supplementary.pdf]

Table S1. Data sumarry for different diets for pediatric and adult patients with IBD.

Diet	Description of Diet	Results in Children	Results in Adults
**Exclusive Enteral Nutrition – EEN (CD)**	Liquid formula alone for 6–8 weeks	Meta-analysis of 8 studies (N = 451) demonstrated similar eficacy as corticosteroids (CS) for induction of clinical and biochemical remission and superiority to CS in terms of mucosal healing [20]	Meta-analysis of 11 studies (N = 378) demonstrated similar clinical remission rates while using elemental, semi - elemental or polymeric formula. Meta-analysis of 8 studies (N = (223) demonstrated similar eficacy as CS for induction of clinical remission. Children achieve higher remission rates than adults (83% vs. 45%) [21]
**Exclusive Enteral Nutrition - EEN (UC)**	Liquid formula + IV corticosteroids (CS) vs. IV CS alone for 7 days		32 patients with acute severe colitis were treated with EEN + IV CS had lower CS failure than the IV CS group, shorter hospital stay, better biochemical markers response, but no difference in colectomy rate [37]
**Exclusive Enteral Nutrition – EEN (UC)**	Liquid formula	15 UC and 24 CD. Shorter time to clinical remission and better weight gain in CD vs. UC [40]	
**Crohn's Disease Exclusion Diet - CDED**	Exclusion of processed food, gluten, milk products and red meat + formula	78 children with mild-moderate CD. Better tolerance than EEN with similar clinical remission rates at week 6. At week 12, CDED plus PEN was effective in sustaining clinical as well as biochemical remission [7].	44 patients with mild-moderate CD.<br>Efficacy has demonstrated in induction of remission as well as maintenance of remission up to 24 weeks [44].
**Partial Enteral Nutrition - PEN**	75% of the caloric intake as formula with 25% as one solid meal	Small study invove 25 children with mildly active CD were divided between EEN (N = 13) and PEN (N = 12). Clinical and endoscopic remission rates at week 6 were comparable between groups [48].	A meta-analysis of 8 studies (N = 429) indicated that PEN helps prevent clinical relapse over 0.5–2 years [49].
**CD-TREAT**	Exclusion of putative pro-inflammatory food components and inclusion of foods high in starch and low in fiber content.	60% clinical remission and 55% reduction in fecal calprotectin after 8 weeks in a pilot study involving 5 children with mild-moderate CD [8].	Similar effects on microbial composition and metabolome as EEN in 25 healthy adults [8].
**Ulcerative Colitis Exclusion Diet (UCED)**	High fiber, low animal fat, saturated and polysaturated fat and low protein as well as exclusion of food additives.	Clinical response and remission in 70.8% and 37.5%, respectively after 6 weeks in 24 children with mild-moderate UC.<br>66% of initial responders remained in remission after 12 weeks [9].	Clinical response of 40% with UCED compared to 11.8% with FMT in a study involving 15 adults with refractory UC. Endoscopic remission was observed in 27% and 11% , respectively [9]
**Specific Carbohydrate Diet (SCD)**	Exclusion of all complex carbohydrates. Monosaccarides are allowed	Clinical remission and normalization of CRP and decreased ESR after 12 weeks in 8 children with mild – moderate CD treated with SCD or modified SCD. Clinical remission was observed also in patients that had more permisive diet [51]	50 adults with IBD reported symptom resolution after a mean duration of 10 months on SCD [54]

		46% of 26 children achieved clinical remission with biochemical response in a retrospective study that included 20 patients with CD and 6 patients with UC [52].	42% of 417 adults reported clinical remission after 6–12 months on SCD [50]
		60% of 10 children with CD achieved clinical remission with a significant decrease in clinical scores [53].	
**Low FODMAP Diet**	Low in fermentable oligosaccharides, disaccharides, monosaccharides and polyols for 4–6 weeks	Beneficial effects on functional gastrointestinal symptoms in 9 children with IBD in remission [62].	Positive effect on gut symptoms in adults with IBD in remission or mild disease [58–60]. Significant decrease in calprotectin levels was demonstrated after 6 weeks in a study that included 55 IBD patients [58].
**IgG4 Guided Exclusion Diet**	Ecxlution of foods that prompt a greater Immunoglobulin (Ig)-G4 response	Not studied in pediatric patients	Improved disease activity scores in adults, though no difference in inflammatory markers was observed in 98 IBD patients with mild activity or in remission [63]
**Anti-inflammatory diet (AID)**	Limitation of certain carbohydrates, increasing intake of pre- and probiotics, unsaturated fats such as omega 3 instead of trans- or polysaturated fats and limiting any specific foods that are not tolerated	Not studied in pediatric patients	Good to very good clinical response reported in 27 IBD patients after 4 weeks and 11 of them had de-escalation of medical therapy [64].
**Mediterranean diet**	Unsaturated and polyunsaturated fats instead of saturated and trans fatty acids, vegetables, whole grains and low intake of red meat	Not studied in pediatric patients	Associassion with reduced liver steatosis, increased quality of life, and lower levels of inflammatory markers after 6 months in 142 IBD patients, with the vast majority of them were in remission at recruitement [67].
			Similar clinical remission rates to SCD, with easier adherence and very limited biochemical resopnse in 191 patients with mild moderate CD after 6 weeks [68].
